# Delayed Presentation of Functioning Metastasis in a Patient with Follicular Thyroid Carcinoma

**DOI:** 10.3390/diagnostics13020281

**Published:** 2023-01-12

**Authors:** Maria Inês Alexandre, Sara Donato, Helena Vilar, Valeriano Leite

**Affiliations:** 1 Serviço de Endocrinologia, Diabetes e Metabolismo, Hospital de Santa Maria, Avenida Prof. Egas Moniz, 1649-028 Lisboa, Portugal; 2 Serviço de Endocrinologia, Instituto Português de Oncologia Lisboa Francisco Gentil, 1099-023 Lisboa, Portugal

**Keywords:** follicular thyroid carcinoma, thyrotoxicosis, metastasis

## Abstract

Functioning metastases from differentiated thyroid carcinoma are rare and present a great therapeutic challenge. Here, we present an unusual case of a patient with metastatic thyroid carcinoma who developed a hyperthyroid state a few years after the diagnosis due to functioning metastases. Radioiodine treatment was effective in controlling the hyperthyroidism; however, it had no effect on tumor burden. By sharing our experience with this case, we hope to raise awareness for this rare condition and the ways to manage it.

**Figure 1:**
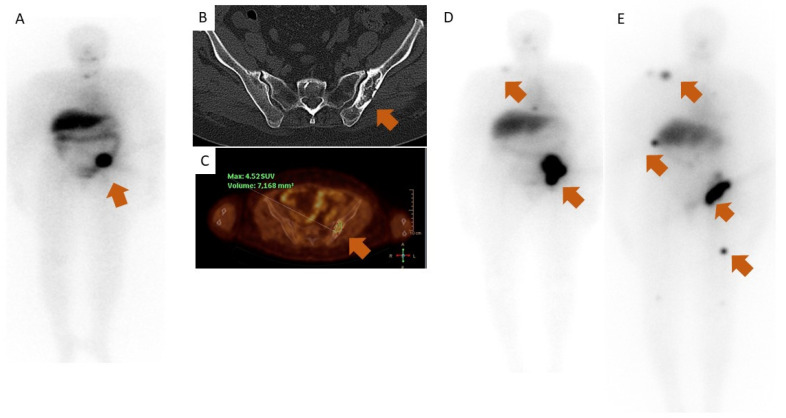
(**A**) Whole-body scintigraphy after ^131^I treatment in 2012 showing tracer uptake (arrow) in the left iliac bone. (**B**) Computed tomography (CT) of pelvis, axial view (the arrow shows the metastasis). (**C**) 18F-fluorodeoxyglucose (18F-FDG)—positron emission tomography (PET) fused with CT. (**D**) Whole-body scintigraphy after the fourth ^131^I treatment in 2018 showing an uptake in the left iliac bone and smaller areas of uptake in the right clavicle and thoracic spine (arrows). (**E**) Whole-body scintigraphy after the sixth ^131^I treatment in 2020 showing an uptake in the left iliac bone and several other areas of smaller uptake (arrows). A 71-year-old woman presented in 2012 with a growing palpable mass in the right side of the neck. She had a past history of a toxic thyroid nodule for which she had been submitted to two treatments with radioactive iodine (^131^I) (total activity of 19 mCi) in 1991. The neck ultrasound revealed an isoechoic solid thyroid nodule with 51 mm, and the fine needle aspiration diagnosed a benign nodule. She underwent right lobectomy followed by total thyroidectomy since histology revealed an oncocytic variant of follicular thyroid carcinoma with extensive vascular invasion. A few weeks later, she was referred to the Nuclear Medicine Department for treatment with 100 mCi of ^131^I. The post-treatment whole-body scan showed increased uptake in the iliac bone compatible with a metastasis, also confirmed by a computed tomography (CT) of the pelvis and a 18F-fluorodeoxyglucose (18F-FDG)—positron emission tomography (PET) scan (**A**–**C**). In the following two years, the patient was submitted to radiotherapy (8 Gy) on the bone metastasis, and to two additional radioactive iodine treatments, both with 150 mCi. During this period, her thyroid hormone levels were well controlled under levothyroxine. In 2018, she presented with symptoms of thyrotoxicosis, namely palpitations, diarrhea and weight loss. Blood tests revealed a suppressed TSH (<0.02 µUI/mL, reference: 0.3–4.2) and elevated triiodothyronine (T3) (346 ng/dL, reference: 80–200), free thyroxine (FT4) (2.22 ng/dL, reference: 0.9–1.7), and thyroglobulin (Tg) levels (4524 ng/mL, reference: <55). Levothyroxine was suspended, and she began treatment with methimazole, 10 mg per day. Thyroid peroxidase antibodies (TPOAb) and thyroglobulin antibodies (TgAb) were negative as well as TSH receptor antibodies (TRAbs). Functioning metastases were suspected, so she was submitted to a fourth ^131^I treatment (150 mCi) in order to control the hyperthyroid state. The post-treatment scan showed the same iliac lesion, with more intense iodine uptake than it was previously documented, as well as new smaller uptake areas in other bones (**D**). After this treatment, she developed hypothyroidism, and serum Tg decreased (1507 ng/mL, reference: <55). She underwent two additional ^131^I treatments, the first in 2019 and the second in 2021, for disease progression and recurrence of thyrotoxicosis. ^131^I was, once again, able to control the thyrotoxicosis, which was difficult to achieve with antithyroid drugs alone. There was also a good response regarding the size of the main lesion in the iliac bone. However, new metastatic lesions were documented after this last treatment (**E**). Due to escalating pain and growth of the known iliac bone metastasis, she also underwent a new radiation treatment (20 Gy). On her most recent follow-up appointment, she maintains disease progression; however, she remains euthyroid under levothyroxine since the last ^131^I treatment. Hyperthyroidism associated with differentiated thyroid carcinoma (DTC) is a rare condition. It may arise from an autonomous functioning thyroid nodule or functioning metastases (FM). A few cases of FM have been reported, in most instances associated with follicular thyroid carcinoma [1,2,3,4,5,6]. Two main mechanisms have been suggested for the pathogenesis of FM: activating mutations of the thyroid stimulating hormone receptors (TSHR) or of the stimulatory guanine nucleotide-binding protein subunit (Gsa), which can lead to constitutive activation of the cyclic adenosine monophosphate-protein kinase A (cAMP-PKA) pathway and result in hyperthyroidism, or overexpression of 5′-iodothyronine deiodinase by the tumor cells, leading to increased conversion of levothyroxine to triiodothyronine (T3). FM present a great therapeutic challenge, as both neoplastic disease as well as thyrotoxicosis must be treated. In most reported cases, thyrotoxicosis is present at diagnosis and, in some cases, it is the initial manifestation of the carcinoma [7,8]. What is particular about this case is that the thyrotoxicosis only presented several years after the initial diagnosis. Hyperthyroidism was present in 1991 when the patient received two treatments with ^131^I and, subsequently, an euthyroid state was induced, which indicates that FM were not present initially. However, disease progression was accompanied by thyrotoxicosis. Both thyrotoxicosis and metastatic lesions increase the morbidity and mortality in patients with FM. Regarding thyrotoxicosis, the usual doses of beta-blockers and antithyroid drugs are generally not helpful. Most patients demonstrate a significant improvement in hyperthyroidism after radioiodine therapy, but in some patients, thyrotoxicosis persists [9]. In this patient, following each radioiodine treatment, there was significant improvement in thyrotoxicosis but there was little effect on the tumor burden. The upcoming challenge in this patient will be the monitoring of the thyroidal function and keeping the patient in euthyroidism so that spontaneous suppression of TSH can be detected while aiming to control disease progression and manage the patient’s symptoms.

## Data Availability

Not applicable.
